# The importance of integrating gender differences in nursing research

**DOI:** 10.4069/whn.2024.12.14

**Published:** 2024-12-30

**Authors:** Jeung-Im Kim

**Affiliations:** School of Nursing, Soonchunhyang University College of Medicine, Cheonan, Korea

## Introduction

Sex is a multidimensional biological construct based on anatomy, physiology, genetics, and hormones, which are sometimes collectively referred to as “sex traits” [1]. The Institute of Medicine emphasizes that these sex traits are fundamental variables in health research, advocating for their consideration at all stages of research in all fields of study [2]. Gender encompasses not only gender identity and expression but also societal and cultural expectations related to these biological traits [3]. Understanding these distinctions is crucial for grasping the full spectrum of human health and disease, impacting everything from diagnosis to treatment; therefore, a precise understanding of these concepts is necessary in healthcare [4].

Research in health sciences has consistently demonstrated that biological and sociocultural factors significantly interact to influence health outcomes [5]. Gender differences affect everything from symptom presentation and disease progression to treatment responses and health outcomes. Neglecting these differences in nursing health research can perpetuate health disparities and diminish the quality of care provided.

Although the importance of sex and gender-based research has been increasingly recognized worldwide, the response from the Korean nursing research community has been less well documented. In a pioneering collaboration, the Korean Society of Nursing Science (KSNS) and Gendered Innovations in Science and Technology Research (GISTeR) have joined forces to spearhead initiatives that bridge the existing gaps in gender-focused research within nursing. This paper outlines the recent efforts driven by this partnership, highlighting the significant strides made in integrating gender perspectives into nursing science and identifying areas that require further exploration.

## First gendered innovation workshop, and forum in nursing research

Since the establishment of the Korean Center for GISTeR in 2016, which became a government-approved foundation in 2021, various seminars have made significant progress in raising awareness of sex and gender differences and gendered innovation. Gendered innovation refers to the integration of sex and gender analysis into all stages of research to improve scientific and technological outcomes, and to ensure that research is more inclusive and addresses the needs of both genders [6]. Textbooks have also been published on gendered innovation in medicine, pharmacology, and the basic sciences. However, it is evident that confusion persists within nursing academia regarding the meaning of sex and gender-based research, and the concepts are not being used appropriately. Recognizing this gap and the general lack of understanding among nurses in Korea, a group of nurse researchers conducted a Gendered Innovation Workshop on September 20, 2024. It featured experts who discussed the historical background and main concepts of gendered innovation and addressed gender issues in caregiving. Following the workshop, KSNS and GISTeR jointly organized the first Gendered Innovation in Nursing Forum on November 5, 2024, opening the door to the field of sex and gender-based research in nursing. The six presentations covered a wide spectrum of topics, such as “Nursing research and gender differences: current status and future directions of gender-reflective nursing research,” “Gender difference in cancer patients,” “Cognitive functions in the elderly and gender differences,” and were streamed via a YouTube channel, attracting over 150 attendees.

## Status of SAGER guidelines in manuscripts

As noted, sex and gender play a pivotal role in determining health outcomes and the Sex and Gender Equity in Research (SAGER) guidelines [6] provide specific guidance on how to address sex and gender in research applying to both humans and animals. The SAGER guidelines have been recognized by many academic institutes and countries and have most recently been adopted by the World Health Organization [7] to set standards for better science. Indeed, adhering to the guidelines is also endorsed by the International Committee of Medical Journal Editors [8]. The following are essential points of the SAGER guidelines:

• Authors should use the terms sex and gender carefully to avoid confusion.

• Where subjects can be differentiated by gender (social and cultural circumstances), the research question should be explored at this additional level of distinction.

• Research on organisms capable of differentiation by sex should be designed and conducted in a way that can reveal sex-related differences in the results, even if not initially expected.

Upon reviewing the SAGER guidelines for nursing journals in Korea, I found that 71.4% of the seven major nursing journals (n=5) specified it in their manuscript guidelines. Although this indicates that a majority of the analyzed nursing journals adhere to these guidelines, nearly one-third have yet to fully integrate these crucial standards.

Five journals, including *Women’s Health Nursing* (WHN) [9], presented detailed descriptions as follows “Ensure correct use of the terms sex (when reporting biological factors) and gender (identity, psychosocial or cultural factors), and, unless inappropriate, report the sex or gender of study participants, the sex of animals or cells, and describe the methods used to determine sex or gender. If the study was done involving an exclusive population, for example in only one sex, authors should justify why, except in obvious cases (e.g., ovarian cancer*). Authors should define how they determined race or ethnicity and justify their relevance (e.g., prostate cancer).”

Two journals, however, gave simpler descriptions, such as “When designing biomedical research, gender variables must be considered”, or “Ensure correct use of the terms sex (when reporting biological factors) and gender (identity, psychosocial or cultural factors), and explain the reason why the gender distribution of the subjects is focused.” Such vague language will need to be made more specific in order for authors and researchers to understand what is necessary.

Thus, not only the difference between sex and gender but also the meaning and interpretation of those characteristics in nursing research, need to be clearly articulated for clarity and adherence.

## Gaps in perceptions of gender differences

As the former editor-in-chief of the *Korean Journal of Women Health Nursing* (the prior title of WHN), I implemented the SAGER guidelines into the instructions for authors. From this perspective, I analyzed how sex and gender have been considered in all studies published in the journal, across all its title variations and without any restrictions on the period of publication. Using the keyword “gender difference,” excluding four editorials, there were only eight papers (Supplementary references) as presented in Table 1. A closer review of articles published in WHN revealed frequent confusion between the terms “sex” and “gender.” Researchers often used the phrase “gender differences” inappropriately, applying it when actually referring to biological sex. This misuse of terms suggests a gap in perception that can potentially inhibit effective communication in research and healthcare policies. This example of published manuscripts in WHN shows that while journal guidelines are increasingly recognizing sex and gender differences, these concepts are often incorrectly described in actual practice.

This discrepancy also alerts us to the need to discuss the implications of these misuses more deeply, such as whether such a nuanced error may affect the study’s outcomes or credibility. As we know, many studies have highlighted sex differences in the epidemiology, pathophysiology, clinical presentation, and outcomes of cardiovascular diseases, osteoporosis, depression and anxiety, drug abuse, and so forth [10-12]. It also challenges authors, reviewers, and journal editors alike to be more sensitive to SAGER and apply accurate descriptions.

## A vision for the future

The lack of comprehensive gender-focused analysis in research not only undermines the validity of studies but also affects the effectiveness of healthcare policies and practices derived from such research. Therefore, integrating a thorough understanding of gender differences into the foundation of nursing research is urgent and necessary. This approach will not only improve the quality of research but also ensure that healthcare practices are equitable and truly responsive to the needs of diverse populations.

To achieve more favorable healthcare outcomes, it is imperative to adopt perspectives that are aligned with the SAGER guidelines and to deepen our understanding of how sex and gender influence health. This knowledge must be systematically integrated into both research methodologies and nursing practices to ensure more effective and inclusive health solutions for everyone. In line with this, we are preparing to publish a textbook on gendered innovation in nursing, aiming to provide a comprehensive resource for the nursing community to promote gender-informed care.

## Conclusion

Despite detailed guidelines, the persistent confusion between sex and gender among researchers indicates a need for better education. This confusion reveals a broader issue: a lack of deep understanding and sensitivity among nursing researchers, including those who review and edit scientific papers. To address this, more targeted education is essential to accurately distinguish and apply the concepts of sex and gender in research. In nursing research, it is imperative to incorporate not only sex characteristics, but also social roles, identities, relationships, and power dynamics associated with gender issues from the research design to the discussion. A textbook on gendered innovation in nursing could provide clear guidance and help establish a standard of care that recognizes unique health needs based on sex and gender. By improving our understanding and integration of gender differences in nursing education and research, we can improve patient care and advance health equity.

## Figures and Tables

**Figure 1.**
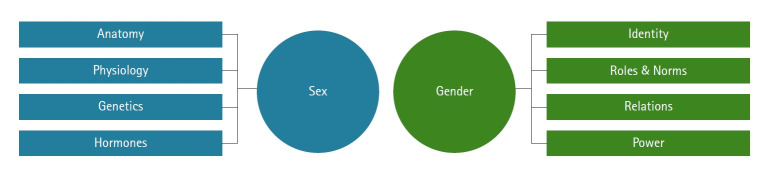
Dimensions of sex (a biological variable) and gender (a social and cultural variable). Adapted from National Institute of Health Office of Research on Women’s Health (https://orwh.od.nih.gov/sex-gender) [4].

**Table 1.** Status of studies referring to sex and gender in *Women’s Health Nursing* (up to the September 2024 issue)

Study	Year	Title	Description	Variables in analysis
Cho and Ahn [S1]	2024	Do age, *gender*, and subjective health-related factors influence health-related life satisfaction in people with disabilities who are physically active?: a secondary analysis	Gender	Male/female, gender
Moon et al. [S2]	2023	Factors affecting the safe sexual behaviors of Korean young adults by *gender*: a structural equation model	Gender	Men/women
Nho and Kim [S3]	2019	*Gender* differences and relationships among lifestyle and reproductive health in university students	Gender	Male/female
Kim and Park [S4]	2015	Relationships among parent-child communication, self-esteem and sexual assertiveness for male and female university students: *gender* difference	Male/female, gender	Male/female
Song et al. [S5]	2015	*Gender* based health inequality and impacting factors	Gender	Man/woman
Kim and Kim [S6]	2014	Sexual behavior and sexual satisfaction according to *gender* in Korean patients with cancer	Gender	Male/female
Cho et al. [S7]	2013	*Gender* differences in awareness of preconception care and pregnancy	Gender	Male/female
Kim [S8]	2011	Comparison of factors associated with intention to receive human papillomavirus vaccine between *male and female* undergraduate students	Male/female	Male/female, gender

The references cited in this table are provided in Supplementary references available online.

## Supplementary materials

Further details on supplementary materials are presented online (available at https://doi.org/10.4069/whn.2024.12.14).

Supplementary references.
