# Supplementary material for: The importance of integrating gender differences in nursing research
Source: Womens Health Nurs. 2024 Dec 30;30(4):254–8. doi: 10.4069/whn.2024.12.14 (PMC11700717; doi:10.4069/whn.2024.12.14)
Supplement: Supplementary references. [file whn-2024-12-14-Supplementary-references.pdf]

Supplementary references

1. Cho H, Ahn S. Do age, gender, and subjective health-related factors influence health-related life satisfaction in people with disabilities who are physically active?: a secondary analysis. Womens Health Nurs. 2024;30(1):56-66. https://doi.org/10.4069/whn.2024.03.18
2. Moon N, Kang H, Heo SJ, Kim JH. Factors affecting the safe sexual behaviors of Korean young adults by gender: a structural equation model. Korean J Women Health Nurs. 2023;29(2):115-127. https://doi.org/10.4069/kjwhn.2023.06.16
3. Nho JH, Kim HS. Gender differences and relationships among lifestyle and reproductive health in university students. Korean J Women Health Nurs. 2019;25(4):446-458. https://doi.org/10.4069/kjwhn.2019.25.4.446
4. Kim BM, Park JS. Relationships among parent-child communication, self-esteem and sexual assertiveness for male and female university students: gender difference. Korean J Women Health Nurs. 2015;21(1):11-22. https://doi.org/10.4069/kjwhn.2015.21.1.11
5. Song MY, Lim WY, Kim JI. Gender based health inequality and impacting factors. Korean J Women Health Nurs. 2015;21(2):150-159. https://doi.org/10.4069/kjwhn.2015.21.2.150
6. Kim JH, Kim HK. Sexual behavior and sexual satisfaction according to gender in Korean patients with cancer. Korean J Women Health Nurs. 2014;20(2):137-147. https://doi.org/10.4069/kjwhn.2014.20.2.137
7. Cho DS, Kim EJ, Jun EM. Gender differences in awareness of preconception care and pregnancy. Korean J Women Health Nurs. 2013;19(4):219-229. https://doi.org/10.4069/kjwhn.2013.19.4.219
8. Kim HW. Comparison of factors associated with intention to receive human papillomavirus vaccine between male and female undergraduate students. Korean J Women Health Nurs. 2011;17(4):415-425. https://doi.org/10.4069/kjwhn.2011.17.4.415
